# Reduction in Hindquarter Vascular Resistance Supports 5-HT_7_ Receptor Mediated Hypotension

**DOI:** 10.3389/fphys.2021.679809

**Published:** 2021-06-24

**Authors:** Bridget M. Seitz, Stephanie W. Watts, Gregory D. Fink

**Affiliations:** Department of Pharmacology and Toxicology, Michigan State University, East Lansing, MI, United States

**Keywords:** 5-HT_7_ receptor, hypotension, hindquarter resistance, rat, hypertension, hemodynamics, regional blood flow, 5-HT

## Abstract

The 5-HT_7_ receptor is the primary mediator of both the *acute* (<hours) and *chronic* (day-week) decreases in mean arterial pressure (MAP) during low dose 5-HT infusion in rats. Previous data show the hypotensive response during chronic 5-HT infusion is due to a decrease total peripheral resistance (TPR) and specifically splanchnic vascular resistance. We hypothesized that changes in vascular resistance in both the splanchnic and skeletal muscle vascular beds are critical to the cardiovascular effects mediated by the 5-HT_7_ receptor. Systemic and regional hemodynamic data were collected in conscious and anesthetized male rats using radiotelemetry, vascular catheters and transit-time flowmetry. Reversible antagonism of the 5-HT_7_ receptor was achieved with the selective antagonist SB269970 (33 μg/kg, iv). From the very beginning and throughout the duration (up to 5 days) of a low dose (25 μg/kg) infusion of 5-HT, TPR, and MAP were decreased while cardiac output (CO) was increased. In a separate group of rats, the contribution of the 5-HT_7_ receptor to the regional hemodynamic response was tested during 5-HT-induced hypertension. The decrease in MAP after 24 h of 5-HT (saline 83 ± 3 vs. 5-HT 72 ± 3 mmHg) was associated with a significant decrease in skeletal muscle vascular resistance (saline 6 ± 0.2 vs. 5-HT 4 ± 0.4 mmHg/min/mL) while splanchnic vascular resistance was similar in 5-HT and saline-treated rats. When SB269970 was administered acutely, MAP and skeletal muscle vascular resistance rapidly increased, whereas splanchnic resistance was unaffected. Our work suggests the most prominent regional hemodynamic response to 5-HT_7_ receptor activation paralleling the fall in MAP is a decrease in skeletal muscle vascular resistance.

## Introduction

A possible role for circulating serotonin (5-HT) in cardiovascular regulation, especially under pathophysiological conditions, is supported by the fact that administration of 5-HT to humans and experimental animals is well-known to affect vascular resistance and arterial pressure (Saxena and Villalon, 1990; Cade et al., 1992; Villalon et al., 2000; Kaumann and Levy, 2006). The magnitude and even direction of these cardiovascular responses are dose-dependent and mediated through several distinct serotonergic receptor subtypes located on vascular smooth muscle, endothelial cells, autonomic nerve endings and in the central nervous system (Noda et al., 2004; Berger et al., 2009). High doses of infused 5-HT cause acute (seconds to minutes) vasoconstriction and pressor responses mediated primarily by 5-HT_2A_ receptors (Hoyer et al., 2002; Calama et al., 2003). Conversely, a low dose of infused 5-HT (25 μg/kg) reduces mean arterial pressure (MAP) acutely and this effect persists over the course of 1–4 weeks of continuous infusion (Diaz et al., 2008; Davis et al., 2012, 2013).

The acute depressor response to 5-HT is mediated via 5-HT_7_ receptors (Terrón, 1997; De Vries et al., 1999; Centurión et al., 2004; Villalon and Centurion, 2007). To confirm the 5-HT_7_ receptor also mediates the chronic (days to weeks) depressor response during low dose infusion, we have shown pharmacological blockade with a selective 5-HT_7_ receptor antagonist, or genetic knockout of 5-HT_7_ receptors, eliminated acute and chronic 5-HT-induced hypotension in conscious rats (Seitz et al., 2017, 2019). These findings strongly support activation of the 5-HT_7_ receptor as critical for the 5-HT-induced depressor response. They provide compelling evidence as to the importance of the 5-HT_7_ in blood pressure regulation, especially in situations where circulating 5-HT levels are modestly increased either acutely or chronically. It is important to note that increased circulating levels of 5-HT in the plasma have been reported in numerous diseases (i.e., carcinoid syndrome, shock, systemic hypertension, pulmonary hypertension, and heart failure (Biondi et al., 1986; Jones and Blackburn, 2002; Ayme-Dietrich et al., 2017). The elevated 5-HT plasma levels in these pathological conditions establishes the importance of understanding how 5-HT influences cardiovascular function chronically and the role of the 5-HT_7_ receptor under those conditions. Here we employed our established low dose 5-HT (25 μg/kg) infusion model (lower dose chosen to mimic modest elevations in plasma 5-HT) to investigate the hemodynamic mechanisms underlying 5-HT induced hypotension.

The hemodynamics which underlies the arterial pressure response elicited by infused 5-HT (5-HT_7_ receptor mediated) are not well understood. Our aim in the present study was to determine the systemic and selected regional hemodynamic effects mediated by the 5-HT_7_ receptor during 5-HT-induced hypotension. With regards to the systemic hemodynamics, MAP is the product of total peripheral resistance (TPR) and cardiac output (CO). TPR mainly reflects arterial smooth muscle tone within the peripheral circulation (Cowley, 1992), whereas CO is mainly determined by cardiac contractility, venous smooth muscle tone, circulatory blood volume and heart rate (Gelman, 2008). Knowing how infusion of 5-HT via activation of the 5-HT_7_ receptor affects these two major hemodynamic measures would help illuminate possible roles of circulating 5-HT under pathophysiological conditions. In addition, from a pharmacological standpoint, any potential therapeutic use of a 5-HT_7_ receptor agonist—for example, in hypertension—would be determined in part by knowledge of the primary systemic hemodynamic target of the drug.

In a previous study, chronic (5-day) 5-HT infusion decreased TPR while CO was increased (Davis et al., 2012). However, these measures were taken only once a day and therefore missed the initial and earlier acute (<24 h) response. Because of that, it is unclear if the observed systemic responses primarily reflect the direct effect of 5-HT_7_ receptor activation or rather time-dependent physiological compensatory responses. Acute hormone actions are apt to be modified under chronic conditions by well-known phenomena such as receptor down-regulation or receptor desensitization. Furthermore, sustained exposure to a vasoactive hormone usually elicits time-dependent physiological compensatory responses (e.g., myogenic responses, stress relaxation, baroreflexes, or pressure natriuresis) which then act to diminish or reinforce the initial response. Specifically, here we suspected the increase in CO we observed was likely due to compensatory rather than direct effects of 5-HT. This was because our previous findings found 5-HT infusion causes dramatic 5-HT_7_ receptor dependent dilation of splanchnic veins (but not arteries) *in vivo* and *in vitro* (Watts et al., 2015; Seitz et al., 2017). Venodilation generally would be expected to cause a fall in CO rather than an increase. In the current study, CO and TPR were calculated both acutely (immediately after starting 5-HT infusion) and chronically (over 5 days) to allow better identification of the direct and indirect hemodynamic responses to 5-HT_7_ receptor activation.

In addition to a vasoactive substance affecting CO by modifying venous smooth muscle tone or cardiac contractility, it can also affect CO simply by redistributing blood flow to the various regional vascular beds, even without changes in venous compliance or capacitance (Coleman et al., 1974; Ogilvie, 1985). This redistribution of blood flow is caused by alterations in various regional vascular resistances which together constitute TPR, the other major determinant of arterial blood pressure.

To more fully define the mechanisms of CO and TPR responses during 5-HT-induced hypotension, we investigated regional hemodynamics in the splanchnic and skeletal muscle vascular beds. The two vascular regions were chosen because they receive a large share of cardiac output (Pang, 2000). Previous evidence suggested that activated 5-HT_7_ receptors in these beds may play a critical role in 5-HT-induced hypotension. For example, in a microsphere study, chronic infusion of low dose 5-HT in conscious rats caused a relatively selective increase in splanchnic blood flow (Seitz and Watts, 2014). Another study using acute 5-HT infusions and microsphere measurements in anesthetized rats (De Vries et al., 1999) found a large increase in skeletal muscle blood flow that was blocked by a 5-HT_7_ receptor antagonist. Thus, both splanchnic and skeletal muscle blood flows were measured in the current study.

## Materials and Methods

### Animals

MSU Institutional Animal Care and Use Committee approved all protocols used in this study. Male Sprague Dawley rats (*n* = 17; 275–300 g; Charles River Laboratories, Portage, MI, United States) were used in the experiments. Rats were singly housed in a temperature–controlled room 22°C with 12-h light/dark cycles and given standard chow and distilled water *ad libitum*. Male rats were used in this study because both male and female Sprague Dawley rats showed similar time and receptor-dependent responses to our established 5-HT infusion model (Seitz et al., 2019). In addition, male and female 5-HT_7_ receptor knockout rats had identical impairments in their cardiovascular response to 5-HT infusion. Due to these findings, males were only used, and we achieved our scientific objective while reducing the number of animals used.

## Systemic Cardiovascular Hemodynamic Measurement Protocol

### Surgical Procedure

Under isoflurane anesthesia (2%) rats were given pre-operative antibiotic (enrofloxacin, 5 mg/kg, im), before being intubated (16-gauge intubation tube) and mechanically ventilated (Kent Scientific Rovent Jr—Rodent Ventilator) on a heated surgical platform to maintain body temperature at 36–37°C. Placement of ascending aorta flow probe (model 2PSB; Transonic System Inc., Ithaca, NY) for measurement of CO was done through a median sternotomy. The flow probe was placed around the ascending aorta just above the coronary arteries. Sterile Surgilube^®^ was used as a coupler to transmit ultrasound signal. The sternotomy was closed in layers and the lungs were reinflated with negative intrathoracic pressure. The flow probe was tunneled subcutaneously and externalized in the mid-scapular region and held in placed with a skin button cuff. The rats were placed in a tethered jacket connected to spring top cage lid to allow for movement around the cage. In the same surgery, a radiotelemeter transmitter (HD-S10; Data Sciences International, MN, United States) for measurement of MAP and heart rate (HR) was implanted subcutaneously through a 1–1.5 cm incision in the left inguinal area. The radiotelemeter catheter tip was introduced into the left femoral artery and advanced into the abdominal aorta below the renal artery. All rats were administered a single dose of sustained release buprenorphine (analgesic; 1.0 mg/kg, sc) and given 7 days of post-operative recovery prior to the start of experiment outlined below.

### Experimental Protocol

**FIGURE 1:**
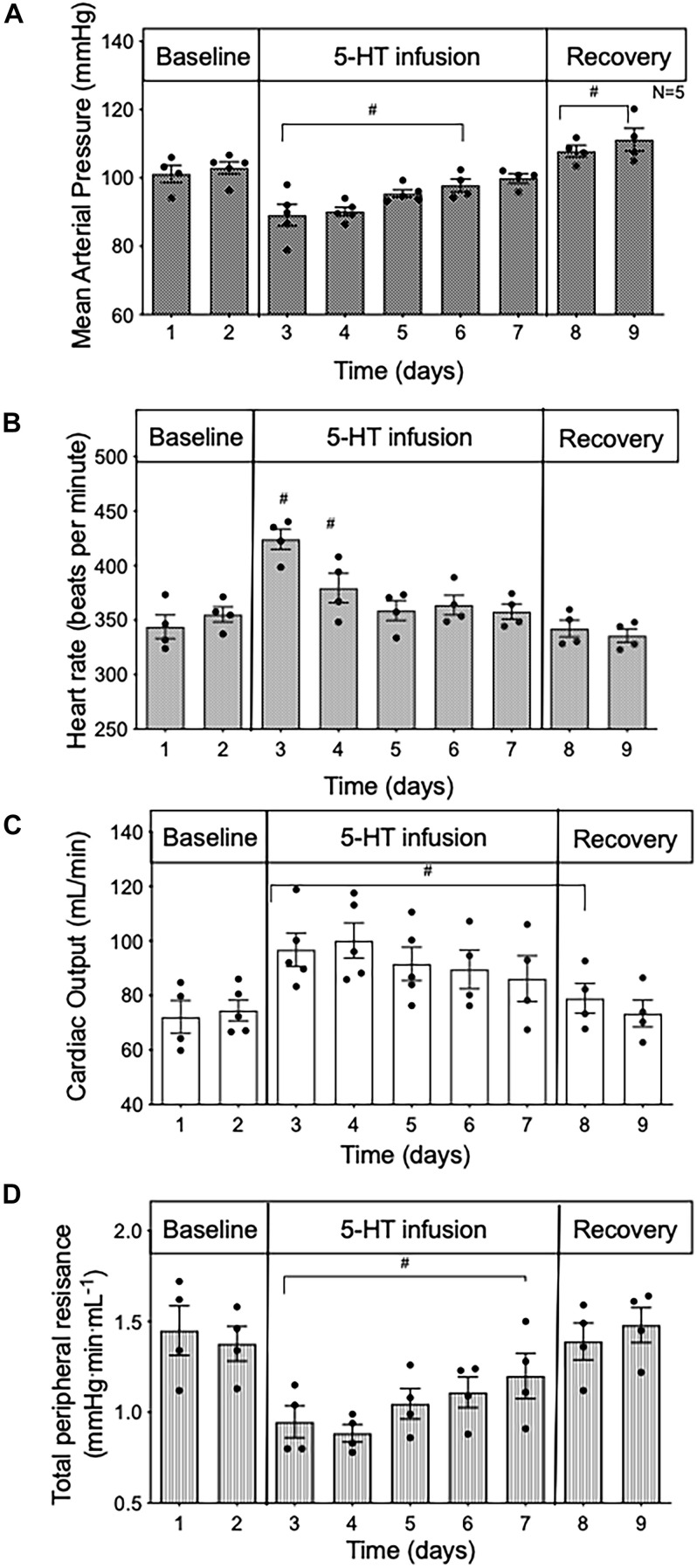
Time course data in conscious rats of **(A)** mean arterial pressure; **(B)** heart rate; **(C)** cardiac output; and **(D)** total peripheral resistance during baseline, 5 days of 5-HT infusion and after 5-HT pumps were removed (recovery) in conscious rats. Data points represent 24 -h averages ± SEM; *n* = 5. A one-way ANOVA with repeated measures was used to determine significance. ^#^*p* < 0.05 compared to baseline. Baseline represents the average of the 2 days prior to the start of 5-HT infusion.

**FIGURE 2:**
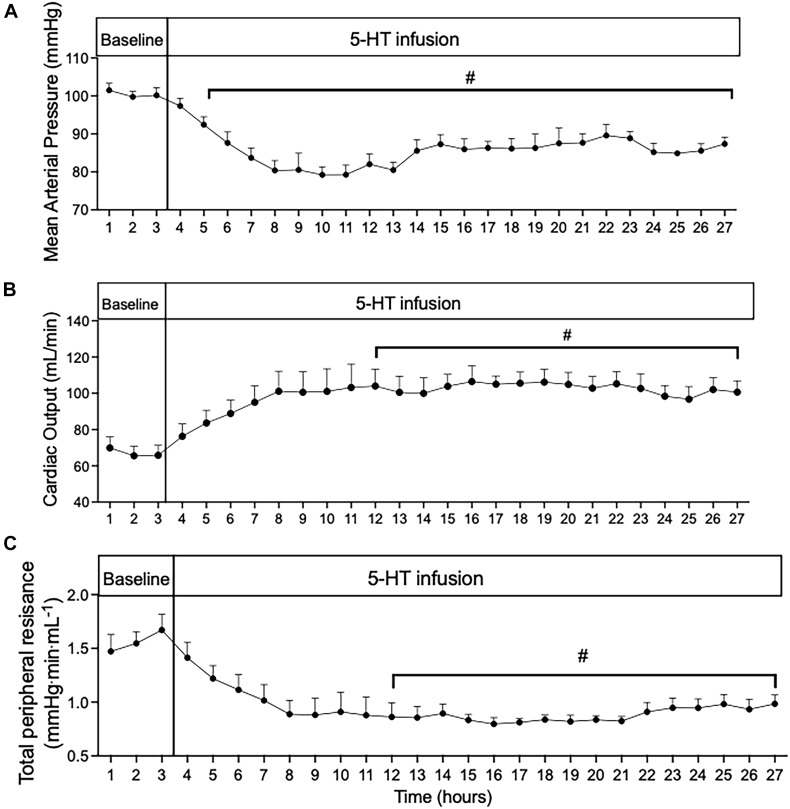
Time course of **(A)** mean arterial pressure; **(B)** cardiac output; and **(C)** total peripheral resistance during 24 h of 5-HT infusion in conscious rats. Data points represent 1-h averages ± SEM; *n* = 5. A one-way ANOVA with repeated measures was used to determine significant differences (^#^*p* < 0.05) compared to baseline. Baseline represents the average of the 3 h prior to the start of 5-HT infusion.

Systemic hemodynamic measurements (MAP, HR, CO, and TPR) were obtained in conscious rats (*n* = 5). Baseline was recorded for 2 days. Osmotic pumps (Model 2ML1; Alzet, Braintree Scientific Inc., Braintree, MA) containing 5-HT (25 μg/kg/min in 1% ascorbate in sterile saline, pH balanced to pH 6–7) were then implanted subcutaneously caudal to the scapular region while under isoflurane anesthesia (1.5%). After 5 days of 5-HT infusion, osmotic pumps were removed under isoflurane anesthesia (1.5%) and cardiovascular parameters were monitored for 2 additional days (termed recovery). The weight of all osmotic pumps was recorded before implantation and after removal to confirm accuracy of drug delivery. The tethered animals were individually housed during all systemic hemodynamic recordings. The chronic study (Figure 1) details 5 days of 5-HT infusion in 24-h intervals. The acute study shown in Figure 2 was taken from the first 24 h from chronic study at the start of 5-HT administration shown in Figure 1. The acute study (Figure 2) details the early systemic response to 5-HT infusion in an hour-by-hour interval. MAP, HR, and CO were continuously measured directly, and data was sent through a data acquisition system (Powerlab, ADI instruments; RRID:SCR_018833). TPR was derived from the following equation: total peripheral resistance = mean arterial pressure/cardiac output.

### Statistical Analyses

Quantitative data are reported as means ± SEM for 5 animals. Statistical analyses were performed using repeated measures one-way ANOVA when comparing systemic hemodynamic response of 5-HT over time with Dunnett’s *post hoc* test for multiple comparisons (GraphPad Prism 8; RRID:SCR_002798). In all cases, a *p*-value of < 0.05 was considered significant.

## Regional Hemodynamic Measurement Protocol

### Surgical Procedure

Under isoflurane anesthesia (2%), rats were instrumented with 3 pressure catheters (polyethylene, ID 03. mm, OD 0.5 mm) for continuous pressure measurements of: (1) MAP via left femoral artery; (2) inferior vena cava (IVC) pressure via the abdominal inferior vena cava by way of the right femoral vein; and (3) portal vein pressure via portal vein by way of the cecal vein. All pressure catheters were attached to a transducer connected to a data acquisition system (Powerlab, ADI instruments) for data collection. In the same surgery, 2 flow probes (Transonic System) were placed to obtain continuous flow measurements of the splanchnic and hindquarter vascular region. For splanchnic vascular flow, a probe (2 mm) was placed around the portal vein (rostral to the portal vein pressure catheter). For hindquarter vascular flow, a probe (1.5 mm) was placed around the terminal end of the abdominal aorta (below the level of the kidneys).

### Experimental Protocol

All animals were anesthetized (2% isoflurane) during this portion of the study to measure the regional hemodynamic responses after 24 h of 5-HT or saline infusion and the contribution of the 5-HT_7_ receptor. Rats were randomized into two groups. Group 1: rats (*n* = 7) were infused with 5-HT (25 μg/kg/min in 1% ascorbate in sterile saline, pH balanced to pH 6–7) pumps and Group 2: rats (*n* = 5) were infused with saline pumps. In all cases, pumps were implanted subcutaneously between the scapulae under isoflurane anesthesia. After 24 h of either 5-HT or saline infusion, the animals were anesthetized and implanted with 3 indwelling pressure catheters and 2 flow probes as described in above surgical section. All rats were given 10 min to stabilize prior to start of baseline measurements. After 10 min of baseline measurements were collected, an acute dose of SB 269970, a 5-HT_7_ receptor antagonist, (33.3 μg/kg) was infused at a rate of 25 μl/min through a separate indwelling right femoral vein line. Splanchnic and hindquarters vascular resistance were calculated using the following equations (Zhang et al., 2013): Splanchnic Resistance = (mean arterial pressure-portal pressure)/portal flow (portal flow is approximately = splanchnic blood flow) and Hindquarters Resistance = (mean arterial pressure-inferior vena cava)/hindquarters flow (hindquarters flow is mostly skeletal muscle/part skin). The portal vein flow was used in the calculation for splanchnic resistance. It is important to note that portal vein flow represents the sum of all the blood entering and leaving the splanchnic vascular bed (except the small amount entering via the hepatic artery). This flow divided by MAP is a reasonable representation of the splanchnic vascular resistance. The hindquarters are made up of mostly skeletal muscle and part skin. In the case of the interpretation of the skeletal muscle resistance, blood flow in the inferior vena cava was used to calculate hindquarters resistance. Data was analyzed at baseline, after 5 min, and 20 min of 5-HT_7_ receptor antagonist administrations in the presence of either 24 h 5-HT or 24-h saline infusion.

### Statistical Analyses

Quantitative data are reported as means ± SEM for number of animals stated above. A two-way ANOVA was used to compare baseline, within groups, and between group regional hemodynamic responses with Tukey’s *post hoc* test for multiple comparisons (GraphPad Prism 8; RRID:SCR_002798). In all cases, a *p*-value of < 0.05 was considered significant.

### Materials

5-HT creatinine sulfate was obtained from Sigma Chemical Company (St. Louis, MO, United States). SB-269970 was purchased from Torcis (R&D, Minneapolis, MN) or Abcam (Cambridge, MA).

## Results

### Systemic Hemodynamics During 5-Day 5-HT Infusion

When averaged over 24 h, MAP (Figure 1A) was reduced during 5-HT infusion, significantly from day 1 to day 4 when compared to baseline values (baseline day one 100 ± 2.4 vs. 5-HT day one 85 ± 1.4 mmHg). In the presence of 5-HT, HR was elevated but only significantly so for the first 2 days of 5-HT delivery (Figure 1B). In contrast, 24-h average CO was significantly increased (Figure 1C), and 24-h average TPR was substantially reduced (Figure 1D) throughout the duration of 5-HT infusion. Once the 5-HT pumps were removed, CO remained elevated for the first day, while MAP rose slightly above baseline levels. TPR returned to baseline values during the recovery phase. These findings confirm those of an earlier study (Davis et al., 2012).

### CO Is Elevated From the Start of 5-HT-Induced Hypotension

In order to gain insight into whether the hemodynamic changes during 5-day 5-HT infusion were a direct effect of 5-HT infusion or primarily compensatory responses, we analyzed the first 24 h of 5-HT infusion in hour-by-hour intervals taken from the chronic experiment shown in Figure 1. From the start of 5-HT administration, the fall in MAP (Figure 2A) occurred in parallel with increased CO (Figure 2B) and decreased TPR (Figure 2C). All variables reached a significant plateau 4–5 h after the start of the infusion. The smooth monotonic increase in CO and decrease in TPR during the first 4–5 h after starting the 5-HT infusion suggests (but doesn’t prove) that the elevation in CO (and decrease in TPR) observed in our chronic 5-HT infusion model was likely caused by direct actions of 5-HT on cardiovascular tissues rather than by time-dependent compensatory responses to those direct actions. Nevertheless, it is clear that compensatory homeostatic responses were engaged during chronic 5-HT infusion. For example, the increases in HR during the first days of infusion were likely mediated via the baroreflex (which then reset over subsequent days. Likewise, the overshoot of mean arterial pressure on the first 2 days after stopping the 5-HT infusion was likely caused by continuing engagement of compensatory pressor mechanisms (e.g., sympathetic activity, renin-angiotensin system, or volume retention) activated during 5-HT induced hypotension.

### Regional Blood Flow Changes Mediated by the 5-HT_7_ Receptor After 24 H 5-HT Infusion

To further understand the cause of the systemic hemodynamics changes we observed, and to characterize the regional hemodynamic effects of 5-HT infusion, we measured: (1) regional blood flow and vascular resistance in the splanchnic and skeletal beds after 24 h of 5-HT infusion; and (2) the contribution of the 5-HT_7_ receptor in the regional response to 5-HT- induced hypotension. We choose to focus on the timepoint of 24 h after starting 5-HT infusion because it is when the greatest fall in MAP occurs in our established model. The measurements were obtained in anesthetized and surgically prepared animals because of the substantial technical difficulties of performing the studies in conscious animals. We are mindful that anesthesia could be a factor when interrupting the data. However, the MAP response in the anesthetized 5-HT animals (unlike what is observed in the anesthetized vehicle-infused animals) were similar to the hypotensive response shown in the conscious 5-HT infused animals and comparable to ours and others published finding (Page and McCubbin, 1953; Dalton et al., 1986; Terrón, 1997; Seitz and Watts, 2014; Seitz et al., 2017).

After 24 h of infusion, MAP was significantly reduced in rats receiving 5-HT compared to saline treated animals (Table 1). Splanchnic blood flow and splanchnic resistance were similar between both 5-HT and saline-treated animals. In contrast, hindquarter resistance was lower (hindquarter blood flow higher) in 5-HT infused vs. saline infused rats (Table 1).

**TABLE 1:** Regional hemodynamic effects after 24 h of 5-HT or saline infusion in anesthetized rats.

	24 h post saline infusion	24 h post 5-HT infusion
MAP (mmHg)	83.4 ± 2.6	71.7 ± 3.3^#^
Portal vein flow (mL/min)	32.7 ± 2.1	29.2 ± 6.3
Splanchnic resistance (mmHg/mL/min)	2.3 ± 0.1	2.6 ± 0.4
Hindquarter flow (mL/min)	14.4 ± 0.6	21.2 ± 2.9^#^
Hindquarter resistance (mmHg/mL/min)	5.5 ± 0.2	3.5 ± 0.4

*Data points represent 24-h averages ± SEM; n = 5–7. A one-way ANOVA with repeated measures was used to determine significance. ^#^p < 0.05 5-HT compared to saline infused rats.*

**FIGURE 3:**
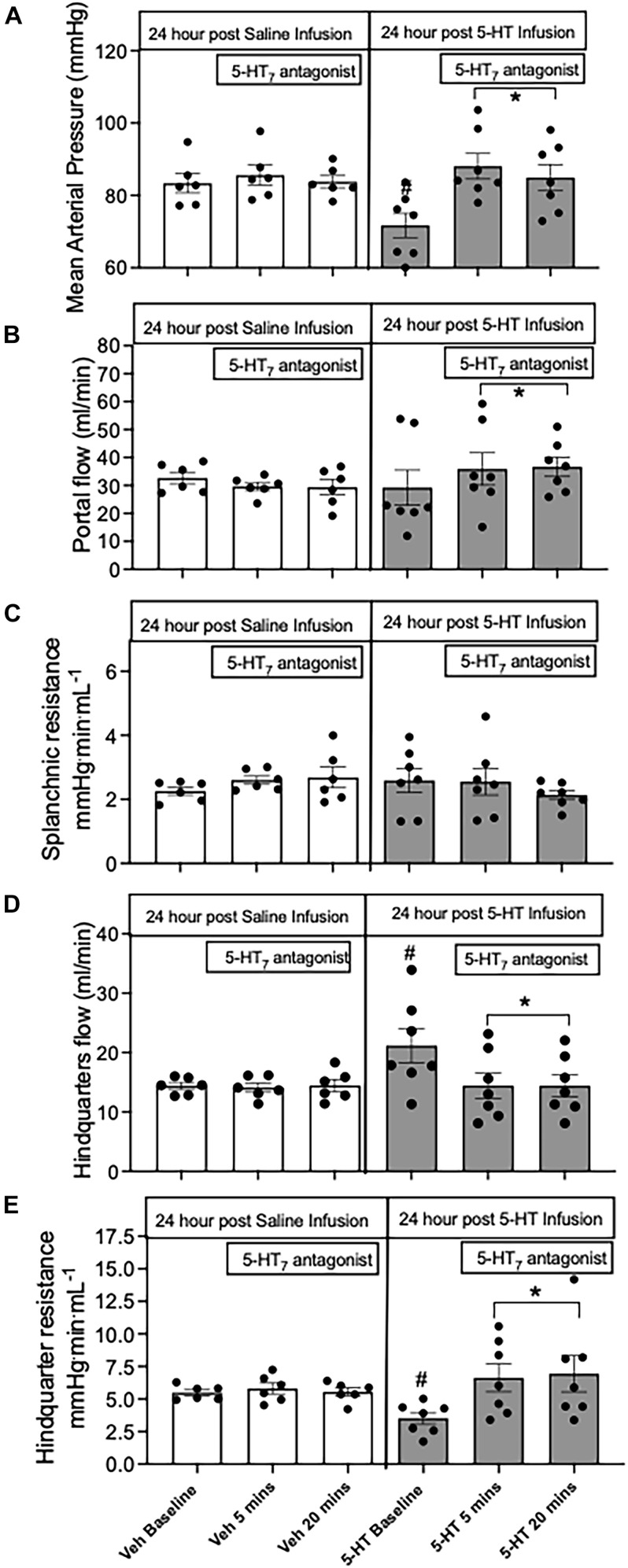
Regional hemodynamic effects mediated by the 5-HT_7_ receptor after 24 h of 5-HT infusion. The hemodynamic effects of **(A)** mean arterial pressure; **(B)** portal vein flow; **(C)** splanchnic resistance; **(D)** hindquarter flow; **(E)** hindquarter resistance after 24 h of infusion of either saline or 5-HT infusion (25 μg/kg/min, osmotic pump) and challenged with the acute 5-HT_7_ receptor antagonist SB269970 (33 μg/kg, iv bolus). Columns represent distinct time points averaged ± SEM: *n* = 5–7 rats. Two-way ANOVA was used to determine significance within groups (*) and between group (^#^). In all cases *p* < 0.05 was significant.

To determine the contribution of the 5-HT_7_ receptor to changes in splanchnic and skeletal muscle vascular resistance after 24 h of 5-HT infusion, an acute bolus of the selective 5-HT_7_ receptor antagonist (SB269970, 33 μg/kg, iv) was given. No significant changes in any regional hemodynamic measurements were observed in rats receiving only saline for 24 h. In rats receiving 5-HT infusion, the decrease in MAP was rapidly restored to near saline–treated levels (Figure 3A) after antagonist administration. Splanchnic blood flow (Figure 3B) increased but this was driven mainly by increased MAP, since splanchnic vascular resistance was unchanged (Figure 3C) by the antagonist. Most notably, in 5-HT infused rats, 5-HT_7_ receptor blockade brought about an immediate increase in hindquarter vascular resistance (Figure 3E) and decrease in hindquarter flow (Figure 3D).

## Discussion

The purpose of this study was to evaluate the systemic and regional (splanchnic and hindquarter) hemodynamic effects of 5-HT_7_ receptor activation in our model of 5-HT-induced hypotension. Our main findings at the systemic level were a reduction in TPR and an increase in CO closely accompanying the fall in MAP. This hemodynamic response pattern occurred at the start (acute; hours) and throughout the duration (chronic; days) of 5-HT infusion. Regarding regional hemodynamics, splanchnic resistance was similar in rats receiving 5-HT compared to saline whereas hindquarter muscle resistance was significantly lower in the 5-HT treated rats. All hemodynamic changes were dependent on 5-HT_7_ receptor activation. These results suggest that a major cause of the hypotension observed in our model of 5-HT infusion is reduced vascular resistance in skeletal muscle (hindquarter).

The focus of this work was predicated on three earlier findings. First, 5-HT infused at 25 μg/kg deceased MAP both acutely (minutes; Page and McCubbin, 1953; Dalton et al., 1986; Terrón, 1997) and chronically (>24 h–month; Diaz et al., 2008; Davis et al., 2012, 2013) when infused into conscious animals. Second, blocking the 5-HT_7_ receptor prevented both the acute (De Vries et al., 1999; Centurión et al., 2004; Villalon and Centurion, 2007) and chronic 5-HT induced depressor response (Seitz et al., 2017, 2019). Finally, and most noteworthy, the same dose 5-HT infused for 1 week reduced arterial pressure in several hypertensive rat models (>30–50 mmHg fall from baseline; Diaz et al., 2008; Watts et al., 2012). This suggests that the activated 5-HT_7_ receptor may be both a critical participant in the cardiovascular effects of endogenously released 5-HT and a potential target for cardiovascular therapeutics.

### Dose of 5-HT to Generate Depressor Response

In our established 5-HT infusion protocol, the dose given produces a concentration of free circulating 5-HT in the plasma that is in the mid-nanomolar range (Diaz et al., 2008). This circulating plasma level of 5-HT is sufficient to activate the 5-HT_7_ receptor (affinity; Ki ∼7 nM), which possesses an affinity approximately two times greater than the other vasorelaxant serotonergic receptors subtypes (5-HT_1B_ ∼14 nM and 5-HT_2B_ ∼19 nM)^1^. Importantly, blockade of the 5-HT_7_ receptor with a selective 5-HT_7_ receptor antagonist SB269970 completely prevented the acute and chronic 5-HT-induced hypotension (Villalon and Centurion, 2007; Seitz et al., 2017). The selective 5-HT_7_ receptor antagonist used in this study and others has an affinity (Ki) for the 5-HT_7_ receptor that is ∼50–1,000 times greater than any other serotonergic receptor subtype (see text footnote 1). More recently, 5-HT_7_ receptor knock-out (KO) rats which we created (Demireva et al., 2019) were given the same low dose of 5-HT for 1 week. The KO rats showed no change in MAP whereas a significant 5-HT-induced depressor response was observed in wild-type littermates (Seitz et al., 2019). These findings indicate that the dose of 5-HT used in our standard infusion protocol, and in the present systemic and regional studies, causes a fall in MAP that is predominately mediated by the 5-HT_7_ receptor under both acute and chronic conditions.

### Systemic Hemodynamic Responses to Low Dose 5-HT

The systemic hemodynamic results to infused 5-HT shown here confirm the findings of our previous study (Davis et al., 2012). The purpose for reinvestigating the systemic hemodynamic response was 2-fold. First, it was to capture the initial acute (<24 h) CO and TPR responses to infused 5-HT. Second, it was to observe the time-course of the return of systemic hemodynamics when the exogenous amine was removed. Neither of these were captured in our earlier study (Davis et al., 2012).

In the current study, TPR was reduced, and CO was increased from the start of 5-HT infusion and throughout the duration of infusion, despite a partial return of MAP toward pre-infusion levels. The decrease in TPR closely mirrored in time the fall in MAP, suggesting that dilation of small arteries and arterioles within the periphery plays a major role in the acute and chronic depressor response to 5-HT. The immediate return of TPR to pre-infusion values after termination of the 5-HT infusion suggests that the change in TPR during infusion is mediated primarily via direct effects of 5-HT_7_ receptor activation.

In contrast, the persistent elevations in CO observed after the 5-HT infusion was stopped suggest that CO is determined at least in part by time-varying compensatory responses to the initial hemodynamic effects of 5-HT. It is important to note that 5-HT is rapidly removed from the circulating plasma by monoamine oxidases found in the liver, or 5-HT transporters found in lung and platelets. These processes maintain low free circulating level of 5-HT in the plasma (∼2% of total body 5-HT), which is estimated to be in the nanomolar range (3–20 ng/mL) (El-Merahbi et al., 2015). Thus, when the 5-HT drug pumps were removed, the circulating level of 5-HT would rapidly (plasma t_1__/__2_ = 1.2 min, Zinner et al., 1983) fall below the level necessary to activate the 5-HT_7_ receptor. This suggests that the 5-HT_7_ receptor has no influence on arterial pressure in the absence of infused 5-HT and that the 5-HT_7_ receptor lacks constitutive activity.

HR was only elevated during the initial 2 days of infusion when MAP was at its nadir. The recovery of HR as MAP increased over several days suggests the observed HR changes are mainly a compensatory baroreflex response than wanes over time due to “resetting.”

We had speculated that the acute (<24 h) response to infused 5-HT would be a decrease in CO along with a decrease in TPR. Our reasoning was based on previous *in vivo* data which showed a low-dose infusion of 5-HT caused a decrease in MAP that paralleled in time a marked increase in the diameter of the large splanchnic veins (portal vein and superior mesenteric vein). Both events were shown to be mediated via the 5-HT_7_ receptor (Seitz et al., 2017). The observed increase in splanchnic vein diameter suggested an increase in venous capacitance and a possible mechanism that could contribute to the 5-HT_7_ receptor induced hypotension, i.e., a fall in CO. However, in the current study, when these variables were measured continuously in a conscious state, 5-HT elicited an increase in CO at the start of infusion. These results suggest any direct effect of 5-HT_7_ receptor activation to increase vascular capacitance – thereby decreasing CO – is overridden by other effects that act to increase CO. Such effects could include increase in sympathetic or decrease in parasympathetic drive to the heart; a reduction in resistance to venous return; or a redistribution of blood flow from high compliance to low compliance compartments of the overall circulation, as described originally by Krogh (1912) and supported by numerous others (Coleman et al., 1974; Greenway, 1981; Ogilvie, 1985).

### Regional Hemodynamic Response to Low Dose 5-HT

To effectively interpret the CO and TPR responses produced in our 5-HT infusion model, we investigated in more detail the hemodynamic response of two regional vascular beds after 24 h of 5-HT or saline infusion. We focused on the splanchnic and hindquarter skeletal muscle regions, as previous evidence supports involvement of these vascular regions in the overall cardiovascular effects of 5-HT_7_ receptor activation (discussed earlier). These regions are generally taken to represent the primary high compliance (splanchnic) and low compliance (skeletal muscle) compartments of the Krogh two-compartment model of the circulation (Krogh, 1912). The kidneys might have been logically included in the regional hemodynamic measurements considering the important contribution of this vascular bed to blood pressure regulation. However, earlier microsphere data indicated a minimal involvement of the renal circulation in acute or chronic 5-HT-induced hypotension (Seitz and Watts, 2014).

In the current study, after 24 h of continuous 5-HT infusion, splanchnic resistances were near baseline values, i.e., comparable to control treated animals. This was the case even though MAP was substantially reduced. This outcome was surprising considering our previous findings with microspheres (Seitz and Watts, 2014), showing a reduction in splanchnic resistance during chronic 5-HT infusion. The reason for the discrepancy is uncertain. Nonetheless, the results suggest some compensatory mechanism must be involved to restore splanchnic resistances during the chronic infusion of 5-HT. There are several possible physiological mechanisms such as inhibition of sympathetic nervous system (Esler, 2000), the hepatic arterial buffer response (Eipel et al., 2010), and release of endogenous vasodilators (nitric oxide, adenosine; Lautt, 2007) that could account for the compensation.

Alternatively, to determine if pharmacological tolerance might explain the changes in hemodynamics after 24 h of 5-HT infusion, we tested the effects of acute blockade of the 5-HT_7_ receptor. This intervention quickly raised MAP in rats infused with 5-HT compared to saline, indicating the 5-HT_7_ receptor was still responsive after 24 h of continuous 5-HT infusion. Receptor blockade had minimal effects on splanchnic resistance (although it declined slightly) even with an increase in portal vein flow. This strongly suggests that the normal splanchnic resistance that was observed in rats after 24 h of 5-HT infusion is mainly due to pharmacological tolerance (loss of 5-HT_7_ receptor activation), perhaps at the level of the splanchnic microcirculation.

The most significant regional vascular response mediated by the 5-HT_7_ receptor was a profound decrease in hindquarter skeletal muscle resistance, such that the increase in skeletal muscle blood flow occurred even in the face of a fall in MAP. Our findings support an earlier microsphere study, where acute 5-HT_7_ receptor-mediated decrease in MAP was associated with a decrease in skeletal muscle vascular resistance (De Vries et al., 1999). The precise location of 5-HT_7_ receptors within the skeletal muscle that mediate the fall in resistance is not known. One previous study observed the small (A3 and A4) but not larger (A1) arterioles from rat cremaster muscle dilated to 5-HT mediated by a 5-HT_7_-like receptor (Alsip and Harris, 1991).

The increase in skeletal muscle flow may help explain the increase in CO observed during 5-HT_7_ receptor mediated hypotension. As described earlier, CO can be strongly affected by the distribution of blood flow (determined by arteries and arterioles) within the various regions of the circulation. The systemic vasculature can be divided into two distinct regions each with a different compliance; low compliance (skeletal muscle and kidney) and high compliance (hepatic-splanchnic) (Krogh, 1912; Coleman et al., 1974; Greenway, 1981; Ogilvie, 1985). *Increasing* blood flow through vascular beds with low compliance, or *decreasing* blood flow through beds with high compliance, will result in an increase in CO even when venous capacitance and compliance are unchanged. Since our results show this response to chronic low dose 5-HT infusion does both, this may provide the necessary and sufficient mechanism for the systemic hemodynamic effects of 5-HT_7_ receptor activation.

## Conclusion

An increase in circulating levels of 5-HT have been shown in cardiovascular disease. Knowing how activation of 5-HT_7_ receptor lowers blood pressure in the presence of elevated 5-HT plasma levels truly highlights the necessity to understand the systemic and regional contributions of this receptor. The experiments described here characterize and analyze the unique systemic and regional hemodynamic effects mediated by the 5-HT_7_ receptor, a member of the 5-HT receptor family whose chronic cardiovascular effects (except for hypotension) have been little studied up to now. Our work reveals novel hemodynamic responses within the splanchnic and skeletal muscle vascular beds. The most substantial response to activation of the 5-HT_7_ receptor paralleling the fall in arterial pressure is an increase in skeletal muscle blood flow by reducing resistance within this region.

## Data Availability Statement

The raw data supporting the conclusions of this article will be made available by the authors, without undue reservation.

## Ethics Statement

The animal study was reviewed and approved by the MSU Institutional Animal Care and Use Committee.

## Author Contributions

BS performed all experiments, analyzed the data, and drafted first version of this manuscript. GF provided experimental oversight, statistical support, and worked on all versions of the manuscript. SW provided initial ideas for work and worked on all versions of this manuscript. All authors contributed to the article and approved the submitted version.

## Conflict of Interest

The authors declare that the research was conducted in the absence of any commercial or financial relationships that could be construed as a potential conflict of interest.

## Footnotes

1 pdsp.unc.edu/databases
